# Measuring body dimensions of leopards (*Panthera pardus*) from camera trap photographs

**DOI:** 10.7717/peerj.7630

**Published:** 2019-09-18

**Authors:** Allan Tarugara, Bruce W. Clegg, Edson Gandiwa, Victor K. Muposhi, Colin M. Wenham

**Affiliations:** 1 Malilangwe Wildlife Reserve, Chiredzi, Masvingo, Zimbabwe; 2 School of Wildlife, Ecology and Conservation, Chinhoyi University of Technology, Chinhoyi, Mashonaland West, Zimbabwe

**Keywords:** Accuracy, Bait, Morphometrics, Non-invasive, Posture

## Abstract

Measurement of body dimensions of carnivores usually requires the chemical immobilization of subjects. This process can be dangerous, costly and potentially harmful to the target individuals. Development of an alternative, inexpensive, and non-invasive method therefore warrants attention. The objective of this study was to test whether it is possible to obtain accurate measurements of body dimensions of leopards from camera trap photographs. A total of 10 leopards (*Panthera pardus*) were captured and collared at Malilangwe Wildlife Reserve, Zimbabwe from May 7 to June 20, 2017 and four body measurements namely shoulder height, head-to-tail, body, and tail length were recorded. The same measurements were taken from 101 scaled photographs of the leopards recorded during a baited-camera trapping (BCT) survey conducted from July 1 to October 22, 2017 and differences from the actual measurements calculated. Generalized Linear Mixed Effects Models were used to determine the effect of type of body measurement, photographic scale, posture, and sex on the accuracy of the photograph-based measurements. Type of body measurement and posture had a significant influence on accuracy. Least squares means of absolute differences between actual and photographic measurements showed that body length in the level back-straight forelimb-parallel tail posture was measured most accurately from photographs (2.0 cm, 95% CI [1.5–2.7 cm]), while head-to-tail dimensions in the arched back-bent forelimb-parallel tail posture were least accurate (8.3 cm, 95% CI [6.1–11.2 cm]). Using the BCT design, we conclude that it is possible to collect accurate morphometric data of leopards from camera trap photographs. Repeat measurements over time can provide researchers with vital body size and growth rate information which may help improve the monitoring and management of species of conservation concern, such as leopards.

## Introduction

Body size is an important variable in carnivore biology. Within populations, individuals of the same species often exhibit variation in body size (Hamilton, 1961; McNab, 2010; Nwaogu et al., 2018). Investigation of the driving factors behind this ecological phenomenon is of conservation and management relevance and consequently, body size has been used to gauge important ecological effects. For example, how body size varies across subjects exposed to different weather conditions (East, 1984; Klein, 1986; Carbone et al., 2014), food resources (Radloff & Du Toit, 2004; Hayward & Kerley, 2008; Owen-Smith & Mills, 2008; Carbone, Pettorelli & Stephens, 2011) and time periods (Jablonski, Erwin & Lipps, 1996; Yom-Tov, 2001; Van Buskirk, Mulvihill & Leberman, 2010). While evaluation of body size may be relatively easy for captive individuals it is more difficult for free-ranging animals. It is especially challenging when subjects are dangerous, for example, leopards (*Panthera pardus*). Under these circumstances, target individuals are usually captured and chemically immobilized. However, this is intrusive, costly and can be potentially harmful to the animals or the handlers (Chinnadurai et al., 2016; Najera et al., 2017). Consequently, it is often difficult to obtain an adequate sample size of body measurements (Fukuda et al., 2013; Law, De Kort & Van Weerd, 2016; Turner et al., 2016; Rothe-Groleau, Rauter & Fawcett, 2018). Body dimensions are routinely measured during collaring exercises but the proportion of collared individuals in each age and sex class is generally low which brings into question the representativity of the morphological data for the different groups (Cichoń, Dubiec & Chadzińska, 2001). Devising a method of remotely obtaining body measurements would improve the resolution of the data because a large proportion of the population could be measured without the need for immobilization.

Little information is available on measuring body dimensions of carnivores from photographs. Ferreira & Funston (2010) and Shumba et al. (2017) successfully measured body dimensions of lions (*Panthera leo*) and wild dogs (*Lycaon pictus*) from photographs collected using hand-held digital cameras. While useful, the method may not be effectively applied to leopards due to their secretive nature. Camera trapping has emerged as a powerful tool for monitoring leopards and similarly marked carnivores in their natural habitats (Karanth & Nichols, 2011; Sollmann, Mohamed & Kelly, 2013; Burton et al., 2015) and the technique could provide a means of addressing this problem. However, measurement of body dimensions of leopards from camera trap photographs has not been attempted. This study seeks to fill this gap.

Here we test a simple method of collecting morphometric data on free-ranging leopards from photographs collected using camera traps in a savanna ecosystem. In this study, baits were used as a means of attracting leopards to camera stations. The main objective of the study was to establish whether it is possible to collect accurate body measurements of leopards from camera trap photographs. The findings may broaden the presently available knowledge on carnivore morphometrics, possibly influencing policy and management, especially where species of interest are hunted. To the best of our knowledge, this is the first study to report on collecting morphometric data of leopards from camera trap photographs in a savanna ecosystem.

## Materials and Methods

**Figure 1:**
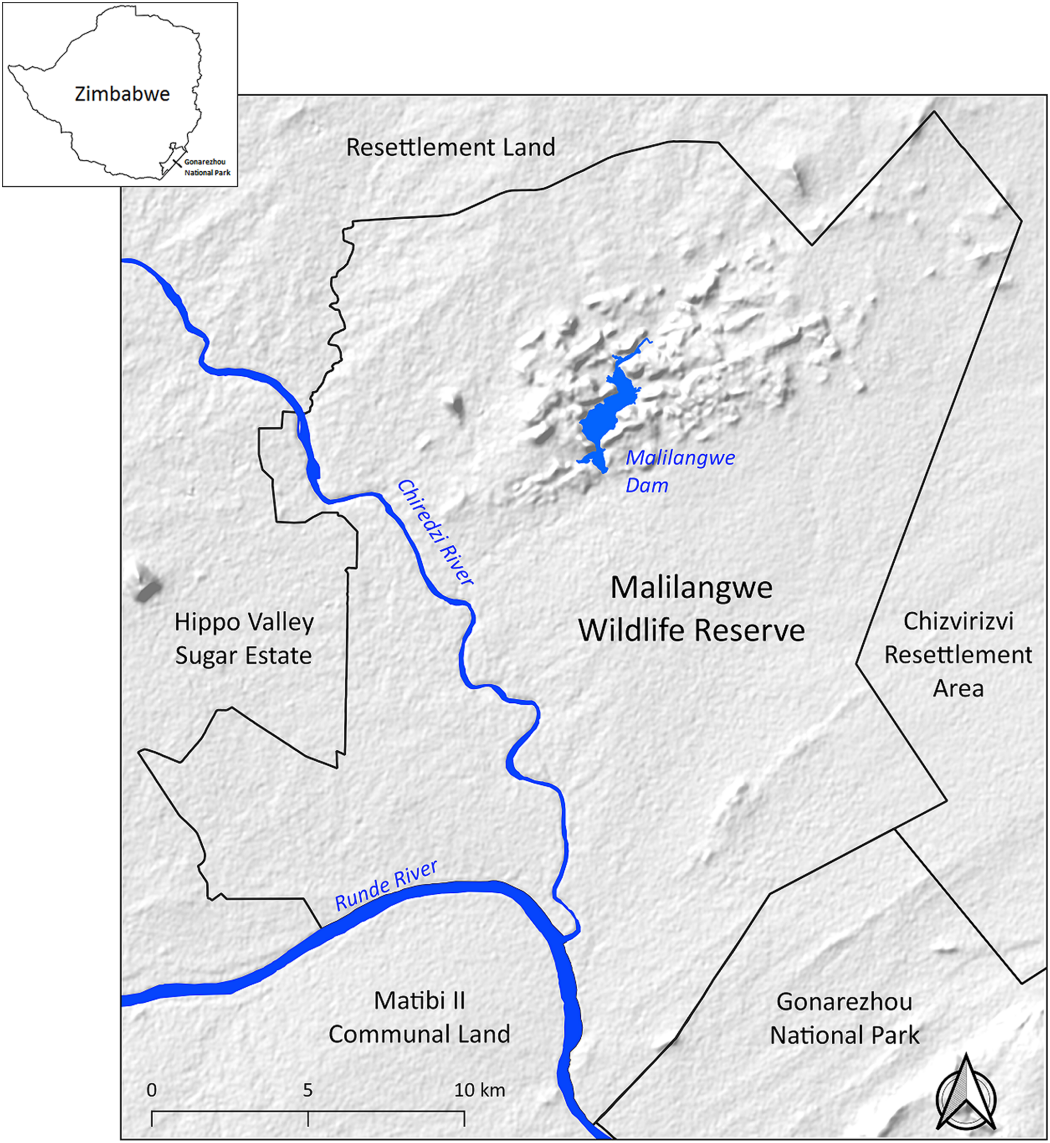
Location of Malilangwe Wildlife Reserve in Zimbabwe.

The study was carried out using a sample of leopards from Malilangwe Wildlife Reserve (MWR), a medium-sized (490 km^2^), fenced, protected area in the semi-arid savanna of south-eastern Zimbabwe (20°58′ and 21°15′S and 31°47′, and 32°01′E) (Fig. 1). MWR is a non-hunting property whose main objectives are conservation and community development. Rainfall (mean ≈ 560 mm per annum, *n* = 66 years, CV = 34%) is seasonal with approximately 84% of precipitation occurring between November and March. The average minimum and maximum monthly temperatures range from 13.4 °C (July) to 23.7 °C (December) and 23.2 °C (June) to 33.9 °C (November), respectively (Clegg & O’Connor, 2017). Altitude ranges from 290 m, in river systems, to 500 m above sea level on sandstone hills (Traill & Bigalke, 2007).

Malilangwe Wildlife Reserve is generally characterized by open savanna woodland dominated by *Colophospermum mopane*. Vegetation cover is diverse, ranging from grassland to dry deciduous woodland, with 38 vegetation types occurring on soils ranging from 90% sand to 40% clay (Clegg & O’Connor, 2012). The leopard population at MWR is estimated at 61 (61–67) individuals (Tarugara et al., 2019) and the main prey species (density in parentheses) are impala (*Aepyceros melampus*, 13.6 km^−2^), nyala (*Tragelaphus angasii*, 0.38 km^−2^) and bushbuck (*T. sylvaticus*, 0.22 km^−2^) (Clegg, 2017). Competing predators include lion (0.1 km^−2^), spotted hyena (*Crocuta crocuta*, 0.12 km^−2^), wild dog (0.06 km^−2^), and cheetah (*Acinonyx jubatus*, 0.02 km^−2^) (Clegg, 2017).

### Research design

In this study, morphometric data were collected in two stages. First, actual dimensions were physically recorded from target individuals chemically immobilized for collaring and second, a set of body measurements from the same collared leopards were obtained from camera trap photographs. Measurements recorded on the leopards themselves served as a baseline upon which comparison with photograph-based measurements could be made. Because it was not logistically possible to obtain reference measurements of the entire leopard population at MWR, photograph-based measurements were limited to sample collared individuals. The dataset comprised measurements taken from multiple photographs of each collared leopard and consequently analysis followed a repeated measures design (Fitt & Lancaster, 2017; Christiansen et al., 2018).

### Data collection

#### Actual body dimensions

Reference measurements were obtained during a leopard collaring exercise conducted at MWR between May 7 and June 20, 2017. Walk in, fall-door traps were used to capture five male and five female leopards for fitting with Followit Global Positioning System collars (Followit, Lindesberg, Sweden). Subjects were chemically immobilized with a combination of Zoletil-Medetomidine (1.0–0.03 mg/kg body mass), with the anesthetic being darted into the muscular region of the hindquarters. Reversal was achieved by injection of Antisedan (at 2.5 mg/mg of Medetomidine) or Yohimbine (at one ml/50 kg of body weight). All handling procedures were performed by a licensed practitioner (with Zimbabwean Dangerous Drugs License number: 2017/25) following safe, professional and humane guidelines (Sikes & Animal Care and Use Committee of the American Society of Mammalogists, 2016). Ethical clearance for the study was granted by the Chinhoyi University of Technology Ethics Committee (clearance number: 01/17).

**Figure 2:**
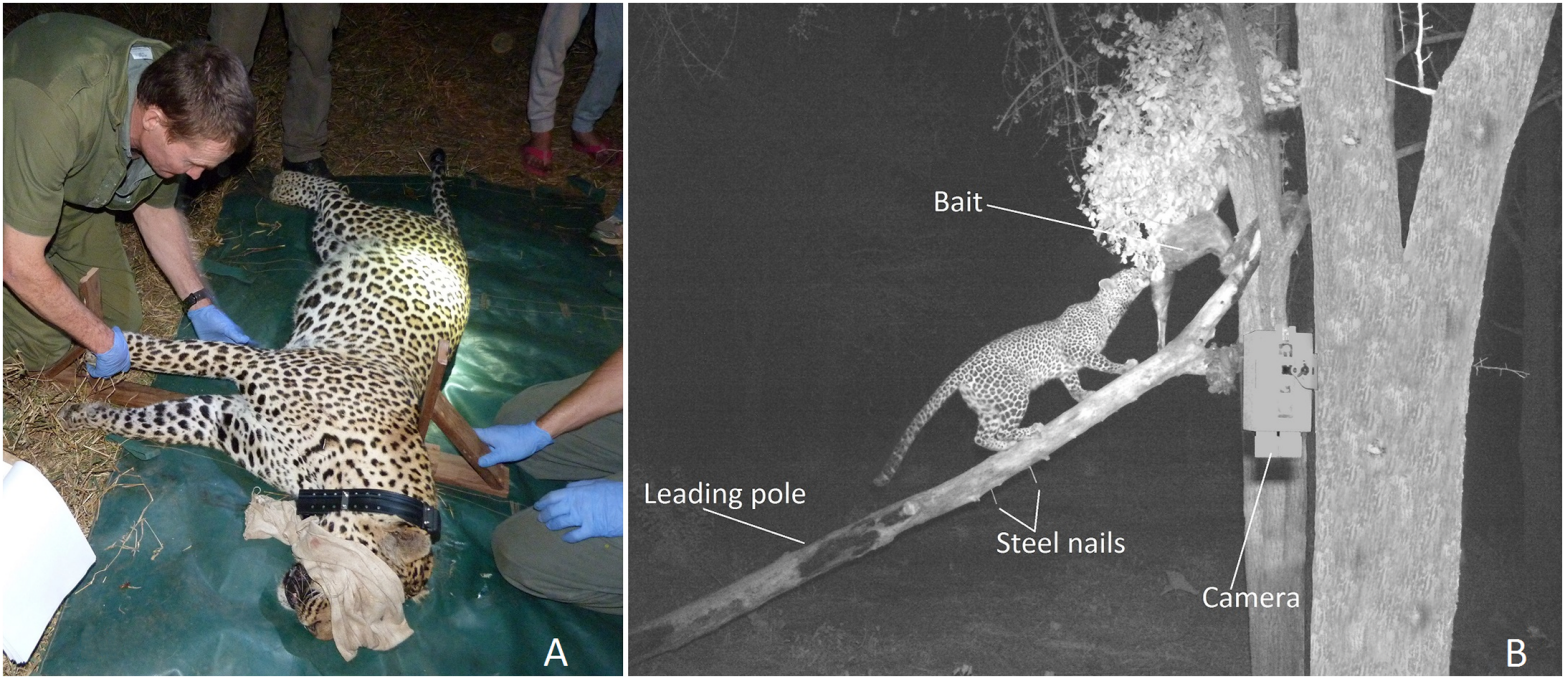
Actual and image-based data collection. (A) Researchers measure shoulder height with a sliding wooden caliper and (B) arrangement of bait, leading pole and camera at sampling stations. (Photo credit: Sarah Clegg).

Following the protocols laid out in De Waal, Combrinck & Borstlap (2004) four morphometric measurements were taken to the nearest 0.1 cm from each anesthetized leopard (Table 1). A non-stretch tape was used to measure body, tail and head-to-tail length and a graduated wooden sliding caliper was devised to record shoulder height (Fig. 2A). A photograph of the right-side profile of each leopard was taken for identification (individual leopards have unique rosette patterns).

**Table 1:** Morphometric measurements recorded for sample leopards.

Measurement	Description
Body length	From the most posterior point of the head along the contour of the body to the proximal base of the tail
Shoulder height	Perpendicular distance between point of shoulder blade to heel of foot
Tail length	From the proximal base to the tip of the last tail vertebra
Head-to-tail length	From the tip of the nose, tracing between the eyes over the head and along the contour of the body to the tip of the tail’s last vertebra

#### Camera trap data collection

Photographic data were collected as part of a baited-camera trapping (BCT) survey conducted in the study area from July 1 to October 22, 2017 (Tarugara et al., 2019). Camera trapping commenced 11 days after collaring so errors due to growth post collaring were negligible. A total of 210 BCT stations were distributed across the study area in a stratified random pattern to record presence data. At each sampling station, two trees spaced two to four m apart were chosen, one for the bait and the other for the camera. An impala carcass was secured to the bait-tree with wire and a leading pole was placed against the tree to provide easy access for leopards. A Cuddeback C2 infra-red camera (Cuddeback, Green Bay, WI, USA) was secured to the camera-tree to the right of each bait. In this way, only the right-side profile of a leopard was photographed. Two stainless steel nails were driven 20 cm apart into the leading pole and cameras set such that photographs included this detail in their frame (Fig. 2B). Hyenas, lions, and elephants (*Loxodonta africana*) visit baited sampling stations, sometimes moving the nails and the leading pole. Photographs without the nails or leading pole were not usable because they could not be scaled. To remedy any interference, sampling stations were monitored every third day and the set-up refreshed.

### Data analyses

#### Photograph-based measurements

Collared individuals were uniquely identified from the rosette patterns on their right flanks. Data for eight of the 10 collared individuals were used in the analyses; one male shrugged off its collar and one female left the reserve early in the study. Photographs containing leopards that were not positioned correctly for measuring were discarded from the dataset before analysis. Data were analyzed using ImageJ software (Schneider, Rasband & Eliceiri, 2012), an image processing program that facilitates scaling and measuring of distances on photographs. A reference measurement was made between the steel nails visible in the photograph. The measurement was taken at the base of the nails (point of entry into the pole) to minimize error resulting from splaying should nails be bumped by animals. By default, ImageJ measures this distance in pixels. The *Set Scale* function of the program was used to define the spatial scale of the photographs such that measurements could be made in calibrated units, for example, centimeters. Because the distance between the camera and the bait-tree could not be standardized, the relative measurement represented by the scaling standard varied between photographs. The known distance (20 cm) between the nails was therefore assigned to the reference measurement and the program calculated a scaling factor (pixels cm^−1^) which was recorded for each photograph. The four body dimensions (shoulder height, head-to-tail, body, and tail length) were measured from the photographs using the same protocols applied when the reference dimensions were recorded. The absolute differences between the ImageJ measurements and the actual recorded during collaring were calculated.

#### Posture categorization

**Figure 3:**
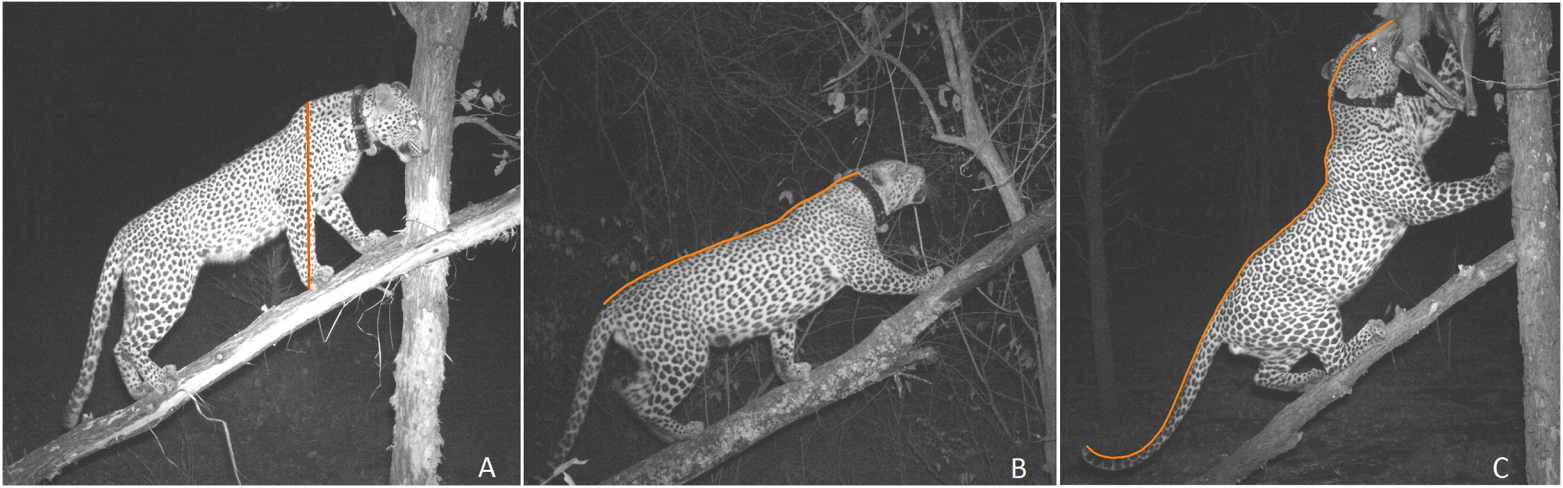
Pictorial representation of three most common leopard postures used in the study. (A) level back-straight forelimb-parallel tail (with outline of shoulder height), (B) level back-bent forelimb-parallel tail (with outline of body length), and (C) arched back-bent forelimb-parallel tail (with outline of head-to-tail length measurement).

Posture was divided into 12 categories (Table 2) and each photograph was assigned the category that best described it (Waite et al., 2007; Meise et al., 2014). Posture categories that had few observations were dropped from the analysis as including them resulted in model convergence issues. The result was a posture variable with three categories (level back-straight forelimb-parallel tail (LB-SF-PT), level back-bent forelimb-parallel tail (LB-BF-PT), and arched back-bent forelimb-parallel tail (AB-BF-PT)) (Fig. 3).

**Table 2:** Posture categories used in the study.

Posture	Description
Level back-straight forelimb-inward tail	Back flat, forelimb extended, tail curved away from observer
Level back-straight forelimb-outward tail	Back flat, forelimb extended, tail curved toward observer
Level back-straight forelimb-parallel tail	Back flat, forelimb extended, tail parallel to leading pole
Level back-bent forelimb-inward tail	Back flat, forelimb angled, tail curved away from observer
Level back-bent forelimb-outward tail	Back flat, forelimb angled, tail curved toward observer
Level back-bent forelimb-parallel tail	Back flat, forelimb angled, tail parallel to leading pole
Arched back-straight forelimb-inward tail	Back contorted, forelimb extended, tail curved away from observer
Arched back-straight forelimb-outward tail	Back contorted, forelimb extended, tail curved toward observer
Arched back-straight forelimb-parallel tail	Back contorted, forelimb extended, tail parallel to leading pole
Arched back-bent forelimb-inward tail	Back contorted, forelimb angled, tail curved away from observer
Arched back-bent forelimb-outward tail	Back contorted, forelimb angled tail curved toward observer
Arched back-bent forelimb-parallel tail	Back contorted, forelimb angled, tail parallel to leading pole

#### Accuracy of measurements

We used the generalized linear mixed-effects model (GLMM) of the *glmer* function in the *lme4* package of R (R Core Team, 2017; Bates et al., 2018) to determine the fixed effects of *body measurement* (factor with four levels), *posture* (factor with three levels), *sex* (factor with two levels) and *scaling factor* on the accuracy of photographic measurements. Multiple measurements of the same leopard or from the same baiting station were not independent, therefore we used *sampling station ID* (spatial non-independence) and *leopard ID* (within subject non-independence) as random effects in the GLMM and the absolute difference between photographic and field measurements as the dependent variable. Distributions plotted using the *ggplot2* package of R (Wickham et al., 2018) showed that data were right skewed and therefore models of the gamma family were constructed, with a loglink function specified to achieve homoscedasticity of residuals. The significance of each fixed effect was determined using a Type II analysis of variance by running the *Anova* function of the *car* package of R (Fox & Weisberg, 2018) on the model’s output. A Type II analysis was chosen because interactions of fixed effects were not specified in the model. The fixed effects of *sex* and *scaling factor* were not significant therefore these were dropped and the model re-run. The *lsmeans* package of R (Lenth, Love & Lenth, 2018) was then used to calculate the least squares means (and 95% confidence intervals) of the absolute differences for the various body measurement and posture combinations. Pairwise comparisons were conducted on the means using Tukey’s post hoc test. A compact letter display was constructed using the *cld* function of the *multcompView* package of R (Piepho, 2004) to show significant differences (α = 0.05) between the least squares means.

## Results

A total of 422 camera trap photographs containing eight of the target leopards, recorded from 26 sampling stations, were retrieved from the global dataset. From these, 101 photographs were used to obtain 210 morphometric measurements (Table 3). All the target leopards used in this study were correctly identified from the photographs. Analysis of variance showed that *body measurement* and *posture* had a significant influence on the accuracy of measurements, while the effects of *sex* and *scaling factor* were not significant (Table 4). The results of the final model run in the GLMM (Absolute difference ~ *body measurement* + *posture* + (1|*leopard ID*) + (1|*sampling station ID*) are given in Table 5.

**Table 3:** Data for fixed effects used in the GLMM. Figures indicate the number of observations per combination of fixed effects and acronyms LB-SF-PT, LB-BF-PT, and AB-BF-PT represent the level back-straight forelimb-parallel tail, level back-bent forelimb-parallel tail and arched back-bent forelimb-parallel tail posture categories, respectively.

Leopard ID	Sex	Body length			Head to tail length			Tail length			Shoulder height	
		LB-SF-PT	LB-BF-PT	AB-BF-PT	LB-SF-PT	LB-BF-PT	AB-BF-PT	LB-SF-PT	LB-BF-PT	AB-BF-PT	LB-SF-PT	LB-BF-PT
Hunyugwe	M	3	3	2	–	1	2	–	1	2	6	2
Mubangweni	M	4	3	4	2	3	4	2	3	4	6	2
Safari camp	M	2	3	–	–	2	–	–	2	–	2	3
Nduna	M	–	2	–	1	1	–	3	2	–	3	3
Chipinyuluzi	F	2	2	1	2	–	2	2	–	1	1	2
Banyini	F	2	9	–	2	10	–	2	9	–	6	4
Swamps	F	1	–	3	1	–	3	1	–	3	1	–
Mamhande	F	5	4	2	4	3	2	4	3	2	14	7

**Table 4:** Analysis of variance for the model: Absolute difference ~ *body measurement* + *scaling factor* + *posture* + *sex* + (1|*leopard* *ID*) + (1|*sampling station ID*).

Fixed effect	d*f*	chisq	Pr (>chisq)
*Body measurement*	3	38.82	<0.001
*Posture*	2	32.61	<0.001
*Sex*	1	1.05	0.306
*Scaling factor*	1	0.12	0.730

**Note:**

Fixed effects with Pr (>chisq) values <0.05 were considered significant.

**Table 5:** GLMM results of the model: Absolute difference ~ *body measurement* + *posture* + (1|*leopard ID*) + (1|*sampling station ID*). LB-BF-PT and LB-SF-PT represent the level back-bent forelimb-parallel tail and level back-straight forelimb-parallel tail posture categories, respectively.

A. Fixed effects	Estimate	Std. Error	*t* value	Pr (>|z|)
*Body measurement*				
Body length (intercept)	1.239	0.167	7.399	<0.001
Head-to-tail length	0.804	0.139	5.787	<0.001
Shoulder height	0.448	0.130	3.439	<0.001
Tail length	0.605	0.138	4.394	<0.001
*Posture*				
LB-BF-PT	0.071	0.160	0.442	0.659
LB-SF-PT	−0.528	0.152	−3.464	<0.001

B. Random effects	Variance	Std.Dev
*Sampling station ID*	0.028	0.169
*Leopard ID*	0.017	0.132
Residual	0.463	0.680

### Accuracy of photograph-based measurements

**Figure 4:**
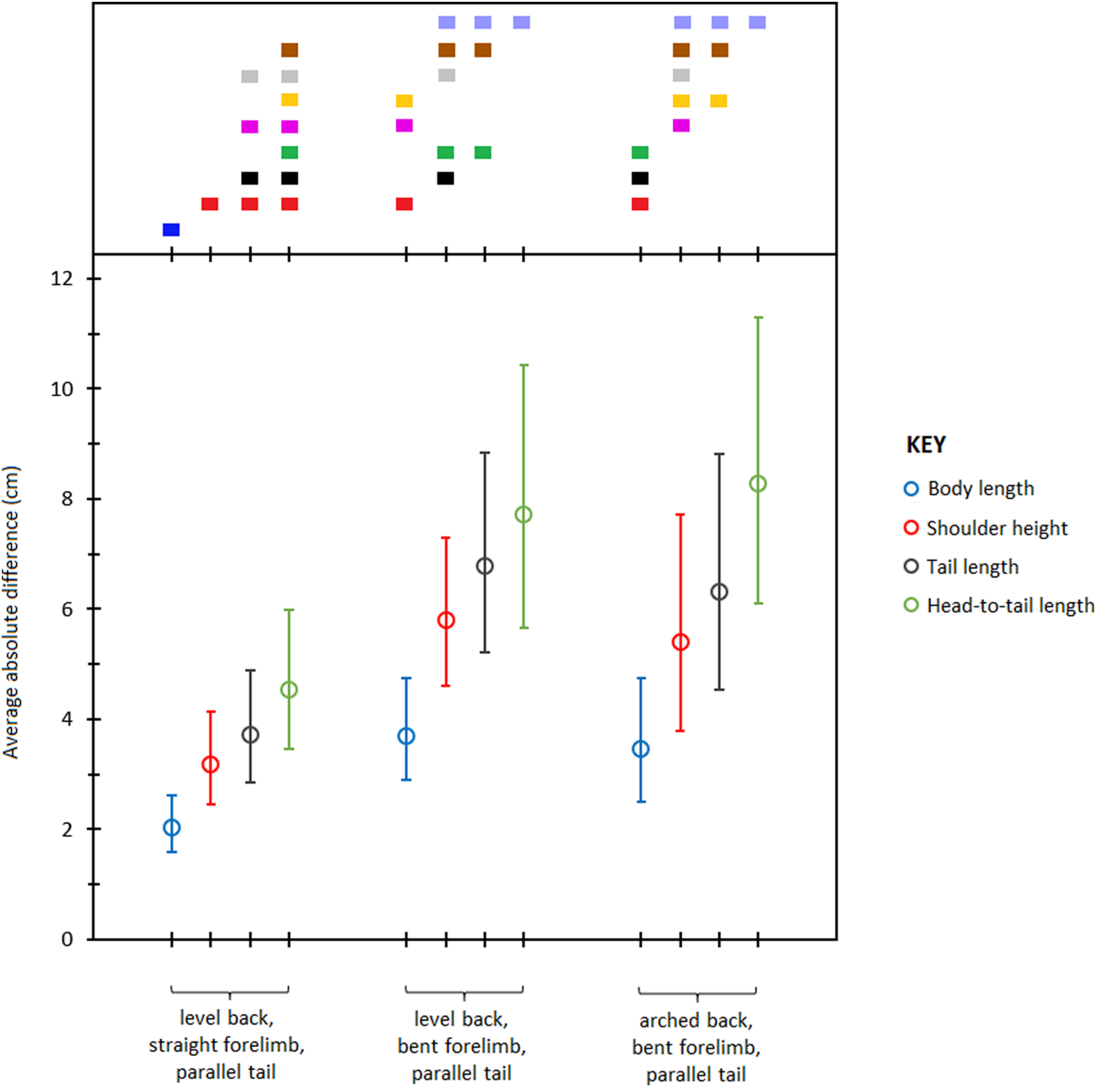
Average absolute differences (least squares means) between photographic and actual dimensions across different body type measurements and postures. Bars represent 95% confidence intervals (back transformed from log scale). The color display (top) depicts the results of pairwise comparisons conducted using Tukey’s post hoc test. Measurements with colors in common were not significantly different (*p* > 0.05).

Pairwise comparisons of least square means of absolute differences (Table 6) showed that body length was measured most accurately from the photographs followed by shoulder height, tail length, and head- to- tail length respectively (Fig 4). Of the three postures, LB-SF-PT produced the most accurate measurements followed by LB-BF-PT and AB-BF-PT respectively. The range of error across the different types of body measurements and postures was 1.5–11.2 cm and the mean scaling factor was 10.4 pixels cm^−1^. Overall, body length measured from the LB-SF-PT posture was most accurate.

**Table 6:** Least squares means and confidence intervals across body measurement and posture categories. Values represent least square means of absolute differences (cm) and 95% confidence intervals (in parentheses). LB-SF-PT, LB-BF-PT, and AB-BF-PT represent the level back-straight forelimb-parallel tail, level back-bent forelimb-parallel tail and arched back-bent forelimb-parallel tail posture categories, respectively.

	LB-SF-PT	LB-BF-PT	AB-BF-PT
Body length	2.0 (1.5–2.7)	3.7 (2.8–4.9)	3.5 (2.5–4.8)
Shoulder height	3.2 (2.5–4.1)	5.8 (4.5–7.5)	5.4 (3.8–7.8)
Tail length	3.7 (2.8–5.0)	6.8 (5.0–9.1)	6.3 (4.5–8.9)
Head-to-tail length	4.5 (3.4–6.1)	7.7 (5.5–10.8)	8.3 (6.1–11.2)

## Discussion

This study demonstrated that it is possible to obtain accurate body measurements of leopards from camera trap photographs in savanna ecosystems. Measurement of body length from the LB-SF-PT posture was the most accurate. The arrangement of material at BCT stations made measurement from photographs possible. Photograph-based measurement of body dimensions has been successfully carried out for primates (Infraorder: *Similiformes*) (Rothman et al., 2008; Barrickman, Schreier & Glander, 2015), sea lions (*Zalophus wollebaeki*) (Meise et al., 2014), western fence lizards (*Sceloporus occidentalis*) (Lambert, Yasuda & Todd, 2012), fish (classes: *Chondrichthyes* and *Osteichthyes*) (Rochet, Cadiou & Trenkel, 2006; Deakos, 2010), horses (*Equus ferus caballus*) (Weisgerber, Medill & McLoughlin, 2015), sheep (*Ovis aries*) (Zhang et al., 2018), and cattle (*Bos taurus*) (Tasdemir et al., 2008; Tasdemir, Urkmez & Inal, 2011). The method described here is best suited for marked carnivores that readily take baits and can climb up the leading pole. With careful consideration, the technique may be modified for studying other carnivore species (e.g., lions, cheetah, and hyenas), viverrids and ungulates provided there is a means of attracting subjects to the site, fixing a scaling standard and ensuring animals are positioned at right angles to the camera’s field of view.

The posture of a leopard in the photograph significantly influenced the accuracy of measurements. Of the three postures, measurements taken from the LB-SF-PT category were the most accurate. This may be because this posture was most consistent with the way in which a leopard was aligned when reference measurements were taken during collaring. A study by Zhang et al. (2018) in China also showed that posture was the main factor influencing accuracy in image-based measurements of sheep. Their study however used a more sophisticated 3-camera design and subjects were photographed in a specialized alleyway. Within postures, measurements of body length were the most accurate followed by shoulder height. This finding was consistent with the work of Tasdemir, Urkmez & Inal (2011) in Turkey and Meise et al. (2014) in the Galapagos Islands who also measured body length accurately in cattle and sea lions using image analysis. Body length provided the most accurate results probably because reference and photograph-based dimensions were taken following the contour of the back (De Waal, Combrinck & Borstlap, 2004) and as such there were no significant differences between the measurements when the back of the leopard was either level or arched. Where leopards are hunted, body measurements of trophy individuals are taken in a straight line that is, placing a taut tape between two pegs marking the extents of a fully stretched out animal (Safari Club International (SCI), 2016). If reference dimensions for this study were taken in this way, variances from the actual may have been larger since leopards seldom assume this posture in photographs.

Compared to body length, the margin of error was greater when measuring shoulder height. This is probably because measurements recorded from the sliding wooden caliper during collaring were taken at 90° to the spinal axis of the leopard. Consequently, deviations from the straight forelimb position would likely affect the accuracy of image-based measurements. This is a limitation of our method since leopards standing on an angled pole may not always align their forelimbs perpendicularly. This may also explain why shoulder height measurements were less accurate than body length. In contrast, shoulder height of lions (Ferreira & Funston, 2010) and wild dogs (Shumba et al., 2017) standing on level ground was measured accurately from photographs.

Across all postures, tail length and head-to-tail length measurements were the least accurate. This may be because the tail and head can articulate independently of the body. The body of a leopard standing on a leading pole was consistently at right angles to the camera’s field of view but the tail and head assumed different angles from photograph to photograph and therefore were not parallel to the leading pole leading to inaccuracies in measurement. Contrary to our findings, Rothman et al. (2008) accurately measured tail length of red colobus monkeys (*Procolobus rufomitratus*) in Uganda from photographs. The greater accuracy in their case might be because the pair of laser points used to calibrate the photographs were projected specifically onto the subjects’ tails, likely reducing the degree of error.

The study showed that sex and scaling factor did not have a significant influence on the accuracy of the image-based measurements. Because multiple photographs recorded at the same station are on a single scale, no significant differences are expected whether male or female leopards are recorded, despite the size difference. The same applies where the same leopard was recorded at various sampling stations and the scaling factors were different. Scaling factor varied from site to site relative to how close or far the subject was in the frame thereby adjusting for measurement error associated with distance from the camera. Similarly, a study conducted in California, USA (Lambert, Yasuda & Todd, 2012) also found that body size or distance of subjects from the camera did not influence the accuracy of morphometric measurements on photographs of western fence lizards collected using a digital camera.

Obtaining full body shots of leopards is crucial for performing measurements. The role of the leading pole at camera stations was central to our study design. The pole ensured that a leopard was positioned at right angles to the camera’s field of view, thus enabling its full body profile to be captured. In conventional (unbaited) camera trapping, unsuspecting leopards are photographed as they pass in front of a camera and as a result the dataset often contains many photographs with frontal, backside and half-body shots (Negroes et al., 2012). In addition, subjects are seldom at right angles to the camera thereby making measurement impossible for such photographs. The pole also ensured that feeding leopards were consistently aligned broadside in a photograph, thus revealing the presence or absence of male external genitalia (Du Preez, Loveridge & Macdonald, 2014). In this way the sex of each individual could easily be determined. If, on the other hand, sex was to be judged based on morphological development, some large females may be mistaken for sub-adult males or vice-versa (Balme, Hunter & Braczkowski, 2012).

To measure distances from a photograph a scaling standard in the frame is required. The leading pole facilitated the easy fixing of steel nails which were visible in the photographs. Other researchers have addressed this issue by modifying digital cameras to project a pair of laser beams onto the subject or objects in the field of view (Rothman et al., 2008; Deakos, 2010; Barrickman, Schreier & Glander, 2015). The distance between the beams is the used to scale the photographs. However, laser products can be potentially harmful to the eyes of animals (Rothman et al., 2008) such that rigging camera sites with lasers may have detrimental effects especially where subjects are likely to stay longer around trapping stations, which is the case with BCT. Furthermore, lasers have been used elsewhere in animal deterrent systems (Gorenzel et al., 2002; Bishop et al., 2003; Akula et al., 2016) and their influence on leopard behavior is not known. Leopards are sometimes startled by shutter sound (A. Tarugara, 2017, personal observation on Moultrie I60 camera traps) and if wary, they might avoid laser-emitting camera traps.

Our findings have demonstrated that photographic measurements of body length recorded from the LB-SF-PT posture can be confidently used to inform on leopard morphometrics. The lowest mean error (2.0 cm for body length in the LB-SF-PT posture) was higher than reported in previous studies (0.2 cm—Bergeron (2007), 1.1 cm—Rothman et al. (2008), 0.75 cm—Willisch, Marreros & Neuhaus (2013)) but was similar to that of distal hindlimb measurements (1.9 cm) in Barrickman, Schreier & Glander (2015). Observed differences between actual and photographic measurements may arise because muscles of immobilized subjects are relaxed while those of active animals are often tensed (Z. Jewell, 2019, personal communication). The ecological significance of this error depends on the intended application. Researchers need to decide whether an estimate of a body measurement and the realized degree of error is sufficiently accurate for their needs or not. For example, a variance of two cm in our case may be sufficiently accurate for monitoring growth over time but may not be sufficiently accurate for individual identification (as two subjects might have similar body sizes).

Morphometric data are important as they broaden our understanding of a species’ biology (Carbone et al., 2014; Codron et al., 2018). Although body measurements can be obtained relatively easily from captive individuals, these data may not be relevant to wild populations due to differences in diet and activity budgets (Altmann & Alberts, 2005; Turner et al., 2016). Body dimensions of wild animals can be measured through capture and chemical immobilization but this often disrupts natural activities and stresses the subjects (Deka et al., 2012). During operations such as collaring and veterinary work, morphometric data are usually collected but these are often ancillary. As a result, sample sizes are usually small and with low representativity. Where remote acquisition of these data is possible, populations can be studied in more detail.

By arranging sampling stations as described above, it is possible to take repeated measurements of individuals non-invasively. The above approach may provide information that can directly inform on growth rates and indirectly on food and habitat quality. Methods that offer accurate and repeatable measurements can be used to monitor growth rates of sample individuals (Rothman et al., 2008) or to model the size structure of populations (Cole, 1994). Where remote collection of morphometric data is possible this enables easier investigation of body size parameters within or among populations (Vindis et al., 2010; Boast et al., 2013; Shumba et al., 2017). For dangerous or shy species, for example, leopards, remote collection of data can be convenient for researchers and subjects alike. In addition, remote measurement is generally less expensive and can likely investigate more subjects compared to physical methods (Bergeron, 2007; Weisgerber, Medill & McLoughlin, 2015). Furthermore, these data can be useful in differentiating sexes in species where size dimorphism is apparent (Marker & Dickman, 2003; Balme, Hunter & Braczkowski, 2012; Farhadinia et al., 2014). Also, where size-minimum harvesting regulations must be observed, morphometric data may have policy and management relevance.

### Future research

Given that morphological development is a function of age (Hilderbrand et al., 2018; Nadal, Ponz & Margalida, 2018), we suggest exploring the possibility of using morphometrics to estimate age of leopards as a next step. This may augment the presently available criteria for aging leopards from photographs developed by Balme, Hunter & Braczkowski (2012) which uses dewlap size, ear condition, facial scarring and nose color as indices. Image analysis has been used elsewhere to estimate body mass in pigs (Brandl & Jørgensen, 1996), cattle (Vindis et al., 2010; Pradana, Hidayat & Darana, 2016) and sea lions (Meise et al., 2014). If software developers could incorporate length, age and weight determination functionalities in camera traps, the range of collectable data would be expanded. For future studies, we suggest fastening the poles and substituting nails with reflective tape or paint as nails are easily bumped by animals.

## Conclusions

Our findings indicate that it is possible to measure morphometric dimensions of leopards from camera trap photographs but the type of body measurement and posture of the target animal are important considerations. We conclude that body length and the LB-SF-PT posture is the combination of choice for accurate measurements. To maximize on the capital investment, we recommend that researchers take advantage of BCT surveys to collect morphometric data for species that are poorly understood.

## Supplemental Information

10.7717/peerj.7630/supp-1 Supplemental Information 1 Raw data used in the GLMM analysis.

10.7717/peerj.7630/supp-2 Supplemental Information 2 R script used for the GLMM analysis.

## Additional Information and Declarations

### Data Availability

The following information was supplied regarding data availability: The raw measurements and the R script used are available in the Supplemental Files.
